# Effects of administration of four different doses of *Escherichia coli* phytase on femur properties of 16-week-old turkeys

**DOI:** 10.1186/s12917-015-0385-x

**Published:** 2015-03-18

**Authors:** Marcin R Tatara, Witold Krupski, Krzysztof Kozłowski, Aleksandra Drażbo, Jan Jankowski

**Affiliations:** Department of Animal Physiology, University of Life Sciences in Lublin, ul. Akademicka 12, 20-950 Lublin, Poland; II Department of Radiology, Medical University of Lublin, ul. Staszica 16, 20-081 Lublin, Poland; Department of Poultry Science, University of Warmia and Mazury in Olsztyn, ul. Oczapowskiego 5, 10-719 Olsztyn, Poland

**Keywords:** Femur, Turkey, Phytase, Volumetric bone mineral density, Skeletal system

## Abstract

**Background:**

The enzyme phytase is able to initiate the release of phosphates from phytic acid, making it available for absorption within gastrointestinal tract and following utilization. The aim of the study was to determine effects of *Escherichia coli* phytase administration on morphological, densitometric and mechanical properties of femur in 16-week-old turkeys. One-day-old BUT Big-6 males were assigned to six weight-matched groups. Turkeys receiving diet with standard phosphorus (P) and calcium (Ca) content belonged to the positive control group (Group I). Negative control group (Group II) consisted of birds fed diet with lowered P and Ca content. Turkeys belonging to the remaining groups have received the same diet as group II but enriched with graded levels of *Escherichia coli* phytase: 125 (Group III), 250 (Group IV), 500 (Group V) and 1000 (Group VI) FTU/kg. At the age of 112 days of life, the final body weights were determined and the turkeys were sacrificed to obtain right femur for analyses. Geometric and densitometric properties of femur were determined using quantitative computed tomography (QCT) technique, while mechanical evaluation was performed in three-point bending test.

**Results:**

Phytase administration increased cross-sectional area, second moment of inertia, mean relative wall thickness, cortical bone mineral density and maximum elastic strength decreasing cortical bone area of femur (P < 0.05). Reduced dietary Ca and P content decreased final body weight of turkeys by 6.5% (P = 0.006). The most advantageous effects of *Escherichia coli* phytase administration on geometric, densitometric and mechanical properties of femur were observed in turkeys receiving 125 and 250 FTU/kg of the diet. Phytase administration at the dosages of 500 and 1000 FTU/kg of the diet improved the final body weight in turkeys.

**Conclusions:**

The results obtained in this study indicate a possible practical application of *Escherichia coli* phytase in turkey feeding to improve skeletal system properties and function.

## Background

Phosphorus (P) is an essential macro-element involved in physiological functions in mammals and birds, including bone tissue formation, energy metabolism, cellular structure, and egg formation. In plant feedstuffs that are commonly fed to growing and laying poultry species, P is primarily stored as phytate P (phytic acid or its salts known as phytates), and is partly unavailable to birds due to lack of sufficient phytase activity [1,2]. Phytates are considered to be anti-nutrient factors which chelate minerals and react with dietary proteins reducing bioavailability of many nutrients [3]. Phytic acid is known to bind calcium (Ca) and P within gastrointestinal tract limiting their intestinal absorption. The enzyme phytase is able to initiate the release of phosphates from phytic acid, making it available for absorption within gastrointestinal tract and following utilization. Phytase-dependent release of the phosphate groups from phytate eliminates the ability of phytic acid to combine with Ca reducing the decline in mineral intestinal absorption [4]. Poultry species do not produce meaningful quantities of endogenous phytase, the enzyme that can hydrolyse the ester bonds between the phosphate groups and the inositol ring in phytates. Thus, routine supplementation of poultry diets with commercially produced phytases is commonplace [5].

Skeletal system disorders in turkeys, especially concerning long bones of legs, are considered as one of the most important factors influencing final production results and economic profits. Economic costs related to leg weakness due to impaired bone growth and development process are significant and result mainly from increased mortality and decreased body weight gain [6]. Fractures occurring in long bones of legs eliminate locomotory functions and encourage subsequent infections. Epidemiological data have shown that mortality in turkey toms due to bone disorders and leg weakness exceeds 3% of the stock [7]. Experimental studies comparing growing turkeys and chickens have shown the highest growth rate of long leg bones in turkeys reaching even 2 mm per day during the first 10 weeks of development [8]. It was confirmed that leg problems, combined with bone development abnormalities occurrence in poultry may be caused by genetic selection for faster growth rate and higher body weight gain, especially when one considers decreased bone mass to muscle mass ratio with increased body weight. Bones of the pelvic limbs overloaded by extremely heavy muscle mass in combination with centre of gravity destabilized by overgrown breast muscles in meat type turkey toms lead to leg deformities and following fractures [9-12]. Experimental studies showing significantly lower incidence of leg abnormalities in turkeys combined with reduced growth rate and body weight gain prove an insufficient adaptation of the skeletal system to rapid development of high muscle mass in meat-type poultry species [13,14].

Considering importance of P bioavailability in growing meat-type turkeys for proper skeletal development and bone tissue homeostasis maintenance, the aim of the study was to determine morphological, densitometric and mechanical properties of femur obtained from 16-week old male turkeys fed with the diet enriched with four different doses of *Escherichia coli* phytase.

## Methods

The study was carried out on the experimental farm (Baldy n. Olsztyn) of the Department of Poultry Science, University of Warmia and Mazury, Olsztyn, Poland. The animal protocol used in this study was approved by the Local Animal Care and Use Committee in Olsztyn, Poland (decision no. 66/2008).

### Experimental design

One-day-old BUT Big-6 male turkeys were delivered to the experimental farm from the commercial hatchery (Hatchery Grelavi Co., Kętrzyn, Poland) and randomly assigned to six weight-matched groups. The turkeys were kept on deep litter at a stocking density of 2.5 bird per m^2^ and eight birds were allocated within one pen. The positive control group (Group I or PC group) consisted of turkeys receiving diet with standard phosphorus (P) and calcium (Ca) content at all feeding stages (Table 1) [15]. The negative control group (Group II or NC group) consisted of birds fed diet with lowered P and Ca content (Table 2) [15]. Turkeys belonging to the remaining groups of the experiment received the same diet as group II but enriched with graded levels of *Escherichia coli* phytase (Optiphos, Huvepharma NV, Belgium): 125 (Group III), 250 (Group IV), 500 (Group V) and 1000 FTU/kg (Group VI). At the age of 112 days of life, the turkeys were weighed and sacrificed to obtain right femur for analyses.

**Table 1:** **Composition and calculated nutritive value of the diet supplied in the positive control group (Group I)**

**Components (%)**	**Age of birds (weeks)**			
	**1 – 4**	**5 – 8**	**9 – 12**	**13 – 16**
Wheat	15.00	15.00	15.00	15.00
Corn	30.12	35.36	39.41	46.35
Soybean meal	43.34	40.19	37.63	28.23
Potato protein	5.00	3.00	-	-
Soybean oil	1.44	1.10	2.00	-
Animal fat	-	1.10	2.13	5.01
NaHCO_3_	0.15	0.10	0.10	0.10
Salt	0.21	0.22	0.20	0.15
Limestone	1.68	1.38	1.23	1.12
MCP	2.00	1.55	1.23	1.05
DL-Methionine	0.31	0.34	0.34	0.33
L-Lysine HCl	0.32	0.29	0.29	0.30
L-Threonine	0.07	0.05	0.13	0.08
Premix (vitamins + trace minerals)	0.25^1^	0.25^1^	0.25^2^	0.25^2^
Choline chloride	0.11	0.08	0.07	0.06
Celite	-	-	-	2.00
**Energy and nutrient content**				
AME_N_ (MJ/kg)	11.61^3^	12.01^3^	12.62^3^	13.22^3^
Crude protein (g/kg)	280^3^/284^4^	255^3^/257^4^	225^3^/231^4^	190^3^/202^4^
Methionine (g/kg)	7.4^3^	7.2^3^	6.6^3^	6.1^3^
Methionine + Cysteine (g/kg)	11.8^3^	11.3^3^	10.4^3^	9.4^3^
Lysine (g/kg)	18.2^3^	16.1^3^	13.9^3^	11.7^3^
Calcium (g/kg)	12.0^3^/12,8^4^	10.0^3^/10.0^4^	8.5^3^/8.4^4^	7.5^3^/7.8^4^
Total phosphorus (g/kg)	8.8^3^/8.7^4^	7.7^3^/7.4^4^	7.0^3^/6.8^4^	6.0^3^/5.9^4^
Available (Av) phosphorus (g/kg)	6.0^3^	5.0^3^	4.3^3^	3.8^3^

^1^Vitamin and mineral premix - nutrients per kg diet: 12,500 IU vitamin A; 5,000 IU vitamin D_3_; 100 IU vitamin E; 4.0 mg vitamin K; 4.5 mg vitamin B_1_;15 mg vitamin B_2_; 5 mg vitamin B_6_; 0.04 mg vitamin B_12_; 110 mg nicotinic acid; 28 mg pantothenic acid; 3.5 mg folic acid; 0.375 mg biotin; 80 mg iron; 25 mg copper; 160 mg manganese; 160 mg zinc; 2.5 mg iodine; 0.3 mg selenium.

^2^Vitamin and mineral premix - nutrients per kg diet: 9,600 IU vitamin A; 4,800 IU vitamin D_3_; 60 IU vitamin E; 3.0 mg vitamin K; 2.0 mg vitamin B_1_;12 mg vitamin B_2_; 5 mg vitamin B_6_; 0.025 mg vitamin B_12_; 85 mg nicotinic acid; 23 mg pantothenic acid; 2.5 mg folic acid; 0.375 mg biotin; 40 mg iron; 25 mg copper; 120 mg manganese; 120 mg zinc; 2 mg iodine; 0.3 mg selenium.

^3^Calculated; ^4^Analyzed [15].

**Table 2:** **Composition and calculated nutritive value of the diet supplied in the negative control group (Group II)**

**Components (%)**	**Age of birds (weeks)**			
	**1 – 4**	**5 – 8**	**9 – 12**	**13 – 16**
Wheat	15.00	15.00	15.00	15.00
Corn	31.84	36.86	41.64	48.43
Soybean meal	43.03	39.82	37.20	27.83
Potato protein	5.00	3.00	-	-
Soybean oil	0.93	1.00	2.00	-
Animal fat	-	1.00	1.38	4.31
NaHCO_3_	0.15	0.10	0.10	0.10
Salt	0.21	0.22	0.20	0.15
Limestone	1.70	1.37	1.18	1.01
MCP	1.09	0.63	0.23	0.18
DL-Methionine	0.31	0.33	0.34	0.32
L-Lysine HCl	0.33	0.29	0.29	0.30
L-Threonine	0.07	0.06	0.13	0.08
Premix (vitamins + trace minerals)	0.25^1^	0.25^1^	0.25^2^	0.25^2^
Choline chloride	0.11	0.08	0.07	0.06
Celite	-	-	-	2.00
**Energy and nutrient contents**				
AME_N_ (MJ/kg)	11.62^3^	12.11^3^	12.62^3^	13.22^3^
Crude protein (g/kg)	280^3^/283^4^	255^3^/263^4^	225^3^/238^4^	190^3^/207^4^
Methionine (g/kg)	7.4^3^	7.2^3^	6.6^3^	6.1^3^
Methionine + Cysteine (g/kg)	11.8^3^	11.3^3^	10.4^3^	9.4^3^
Lysine (g/kg)	18.2^3^	16.1^3^	13.9^3^	11.7^3^
Calcium (g/kg)	10.1^3^/10.5^4^	8.0^3^/7.9^4^	6.5^3^/6.3^4^	5.5^3^/5.4^4^
Total phosphorus (g/kg)	6.8^3^/7.0^4^	5.7^3^/5.8^4^	4.8^3^/4.9^4^	4.1^3^/4.1^4^
Available (Av) phosphorus (g/kg)	4.0^3^	3.0^3^	2.1^3^	1.9^3^

^1^Vitamin and mineral premix - nutrients per kg diet: 12,500 IU vitamin A; 5,000 IU vitamin D_3_; 100 IU vitamin E; 4.0 mg vitamin K; 4.5 mg vitamin B_1_;15 mg vitamin B_2_; 5 mg vitamin B_6_; 0.04 mg vitamin B_12_; 110 mg nicotinic acid; 28 mg pantothenic acid; 3.5 mg folic acid; 0.375 mg biotin; 80 mg iron; 25 mg copper; 160 mg manganese; 160 mg zinc; 2.5 mg iodine; 0.3 mg selenium.

^2^Vitamin and mineral premix - nutrients per kg diet: 9,600 IU vitamin A; 4,800 IU vitamin D_3_; 60 IU vitamin E; 3.0 mg vitamin K; 2.0 mg vitamin B_1_;12 mg vitamin B_2_; 5 mg vitamin B_6_; 0.025 mg vitamin B_12_; 85 mg nicotinic acid; 23 mg pantothenic acid; 2.5 mg folic acid; 0.375 mg biotin; 40 mg iron; 25 mg copper; 120 mg manganese; 120 mg zinc; 2 mg iodine; 0.3 mg selenium.

^3^Calculated; ^4^Analyzed [15].

### Bone analysis

The right femur was isolated and cleaned of all soft tissues to determine weight, length, volumetric bone mineral density (vBMD), and morphometric and mechanical properties of the bone. After isolation, the bone samples were frozen in plastic bags and kept at −25°C until further analyses. Volumetric bone mineral density of each femur was measured with the use of quantitative computed tomography (QCT) technique and Somatom Emotion Siemens apparatus supplied with Somaris/5 VB10B software (Siemens, Erlangen, Germany). Prior to scanning procedure, bones were thawed in bags for 2 hours at room temperature. Raw data acquisition using the scanning procedure lasted approximately 10 minutes for 8 bones representing single experimental group. The measurement of vBMD was performed for the trabecular and cortical bone compartments using 2-mm thick cross-sectional metaphyseal and diaphyseal QCT scans. Volumetric BMD of the trabecular bone (trabecular bone mineral density – Td) of femur was determined in the distal metaphysis of femur at 7% of total bone length, measuring from the distal extremity. Volumetric BMD of the cortical bone (cortical bone mineral density – Cd) was measured on mid-diaphyseal scan placed at 50% of the femur length. Cortical bone area (CBA) was measured automatically at the midshaft of femur. Volume evaluation software (Siemens, Erlangen, Germany) was used to measure the total bone volume (Bvol) and mean volumetric bone mineral density (MvBMD) of each femur. For Bvol and MvBMD measurements, the volume-of-interest was limited by minimum and maximum density of the investigated samples at 0 and 3000 Hounsfield units, respectively. The measurements of Bvol and MvBMD were executed for the whole bone, and results obtained reflect the values determined within all anatomic structures of the investigated bone.

Geometrical properties of each femur were determined on the basis of measurements of horizontal and vertical diameters of the mid-diaphyseal cross-section of the bone obtained from computed tomography multiplanar reconstructions. The values of cross-sectional area (A), second moment of inertia (Ix), mean relative wall thickness (MRWT) and cortical index (CI) were estimated.

Mechanical properties of femur were determined using the three-point bending test in Instron 3367 apparatus (Instron, Canton, MA, USA) combined with a computer. The relationship between force perpendicular to the longitudinal axis of the bone and the resulting displacement was presented graphically, and the values of maximum elastic strength (Wy) and the ultimate strength (Wf) were determined. The distance between supports of the bone was set at 40% of total femur length and the measuring head loaded bone samples at the midshaft with a constant speed of 50 mm/min.

### Statistical analysis

All data were expressed as means and ± S.E.M. The results obtained were analyzed with a use of one-way analysis of variance (ANOVA) and Statistica software v. 10.0 PL. Statistical significance of the differences of the investigated variables between the groups was determined using post hoc multiple comparisons Duncan’s test. For all comparisons, P-value ≤ 0.05 was considered as statistically significant.

## Results

### Body weight

Initial and final body weights of turkeys are shown in Table 3. At the beginning of the experiment, initial body weights of turkeys from all the groups were not significantly different (P > 0.05). Final body weight of 112-day old turkeys from the negative control group was significantly lower when compared to the values in the positive control group (P = 0.006) and groups V and VI receiving the diet enriched with 500 and 1000 FTU/kg of *Escherichia coli* phytase (P < 0.05).

**Table 3:** **Initial and final body weight of turkeys from the positive and negative control groups (Group I and Group II), and the groups receiving diet experimentally enriched with 125 (Group III), 250 (Group IV), 500 (Group V) and 1000 (Group VI) FTU/kg of** ***Escherichia coli*** **phytase**

**Investigated parameter**	**Group I (PC group; n = 8)**	**Group II (NC group; n = 8)**	**Group III (n = 8)**	**Group IV (n = 8)**	**Group V (n = 8)**	**Group VI (n = 8)**
Initial body weight (g)	59.56 ± 0.71	59.42 ± 0.37	60.04 ± 0.86	59.62 ± 0.73	59.62 ± 0.69	59.42 ± 0.64
Final body weight (g)	14463 ± 680^a^	13525 ± 755^b^	13988 ± 461^ab^	14054 ± 595^ab^	14201 ± 460^a^	14380 ± 563^a^

Values are means ± SEM.

^ab^Statistically significant differences between the groups are indicated with different superscript letters for P ≤ 0.05.

### Bone properties in 112-day-old turkeys

Morphometric, densitometric and mechanical parameters of femur in 112-day old turkeys are shown in Table 4. Bone weight reached significantly higher values in groups V and VI when compared to groups II and IV (P < 0.05). Bone length was significantly higher in group V when compared to the values obtained in groups I, II, III and IV (P < 0.05). Total bone volume was significantly higher in group V when compared to group IV (P = 0.04). Cortical bone area was significantly higher by 9.3% in the positive control group when compared to the negative control group (P = 0.02). The values of cortical bone area were significantly decreased in groups III, IV, V and VI when compared to groups I and II (P < 0.05). Cross-sectional area was found to be significantly higher in group III when compared to the values of this parameter obtained in all other groups of the experiment (P ≤ 0.05). Second moment of inertia (Ix) reached the highest value in group III, and that was significantly different than the values in group I, II, IV and V (P < 0.05). The value of Ix obtained in group VI was significantly higher when compared to groups I, II and IV (P ≤ 0.01). Statistically higher value of Ix was found in group V when compared to group II (P = 0.02). The values of MRWT and CI reached significantly higher values in group III when compared to all other groups of the experiment (P < 0.05). Trabecular bone density was significantly higher by 7.2% in group III when compared to group IV (P = 0.03). Volumetric BMD of cortical bone was significantly higher in groups IV and V when compared to the positive and negative control groups (P < 0.05). Moreover, Cd values in groups III and VI were significantly higher when compared to the values obtained in the negative control group (P < 0.05). Maximum elastic strength of femur was significantly higher in groups III and IV when compared to the values obtained in groups I and VI (P ≤ 0.05). The values of the ultimate strength obtained in all the groups of experiment (I – VI) were not found to be significantly different (P > 0.05).

**Table 4:** **Morphometric, densitometric and mechanical properties of femur in 112-day old turkeys from the positive and negative control groups (Group I and Group II), and the groups receiving diet experimentally enriched with 125 (Group III), 250 (Group IV), 500 (Group V) and 1000 (Group VI) FTU/kg of** ***Escherichia coli*** **phytase**

**Investigated parameter**	**Group I (PC group; n = 8)**	**Group II (NC group; n = 8)**	**Group III (n = 8)**	**Group IV (n = 8)**	**Group V (n = 8)**	**Group VI (n = 8)**
Bone weight (g)	37.41 ± 0.91^ab^	34.44 ± 1.34^b^	37.89 ± 1.64^ab^	34.49 ± 0.62^b^	39.58 ± 1.70^a^	38.84 ± 0.86^a^
Bone length (mm)	145.4 ± 1.0^a^	146 ± 1.15^a^	145.9 ± 1.1^a^	146.1 ± 0.9^a^	149.8 ± 1.7^b^	148.4 ± 1.0^ab^
Bone volume (cm^3^)	17.27 ± 0.74^ab^	15.62 ± 1.00^ab^	17.17 ± 1.13^ab^	15.20 ± 0.37^a^	18.06 ± 1.06^b^	17.52 ± 0.7^ab^
Cortical bone area (mm^2^)	89.0 ± 2.4^a^	80.75 ± 2.8^b^	72.6 ± 2.5^c^	70.3 ± 1.5^c^	69.5 ± 1.7^c^	71.3 ± 3.2^c^
Cross-sectional area (mm^2^)	77.1 ± 3.5^a^	76.3 ± 4.3^a^	100.2 ± 4.3^b^	79.5 ± 2.8^a^	83.7 ± 2.4^a^	88.7 ± 6.4^a^
Second moment of inertia (mm^4^)	2476 ± 177^ab^	2029 ± 148^a^	3338 ± 254^c^	2269 ± 105^ad^	2703 ± 133^bde^	3172 ± 258^ce^
Mean relative wall thickness	0.213 ± 0.010^a^	0.234 ± 0.016^a^	0.269 ± 0.007^b^	0.220 ± 0.011^a^	0.217 ± 0.007^a^	0.206 ± 0.016^a^
Cortical index	17.5 ± 0.6^a^	18.8 ± 1.0^a^	21.1 ± 0.4^b^	17.9 ± 0.7^a^	17.8 ± 0.4^a^	16.9 ± 1.1^a^
Trabecular bone density (g/cm^3^)	0.839 ± 0.028^ab^	0.844 ± 0.019^ab^	0.893 ± 0.021^a^	0.823 ± 0.015^b^	0.847 ± 0.017^ab^	0.854 ± 0.015^ab^
Cortical bone density (g/cm^3^)	1.967 ± 0.060^ab^	1.925 ± 0.040^a^	2.065 ± 0.037^bc^	2.145 ± 0.048^c^	2.135 ± 0.039^c^	2.091 ± 0.043^bc^
Mean volumetric bone mineral density (g/cm^3^)	1.592 ± 0.019	1.546 ± 0.019	1.556 ± 0.031	1.595 ± 0.023	1.575 ± 0.021	1.543 ± 0.018
Maximum elastic strength (N)	242 ± 14^a^	305 ± 21^ab^	330 ± 21^b^	350 ± 30^b^	295 ± 11^ab^	266 ± 26^a^
Ultimate strength (N)	526 ± 42	576 ± 35	586 ± 28	575 ± 43	569 ± 40	493 ± 44

Values are means ± SEM.

^a-e^Statistically significant differences between the groups are indicated with different superscript letters for P ≤ 0.05.

## Discussion

Numerous techniques may be used for skeletal system quality evaluation in poultry species. Postmortal isolation of bone samples enables macro-morphological evaluation in terms of bone weight, length, thickness of compact and spongy layers, and perimeters and diameters of diaphysis, epiphyses and metaphyses [16]. Microscopic evaluation of histological preparations may be used for investigation of endochondral ossification process in growth plates [17]. Microradiographic imaging of transverse sections of bone shaft is utilized for cortical porosity evaluation and standard radiographic mineral density measurement [18]. Dual-energy X-ray absorptiometry (DEXA) method and digital analysis of radiograms using precise software such as Trabecula® may be applied for both *in vivo* and *ex vivo* assessment of bone mineral density, bone mineral content and microarchitectural organization of bone samples [19,20]. As opposed to the mentioned above diagnostic techniques which provide two-dimensional analysis of bone samples, quantitative computed tomography enabling three-dimensional morphological and densitometric analysis of bones was used in this study [21].

Pelvic limb long bones such as femur, tibia and tibiotarsus in bipedal domestic meat-type birds are subjected to significant bending forces in order to withstand loading by body tissues [22-24]. It was reported that incidence of clinical manifestations of metabolic bone disorders such as leg deformations, lameness and bone fractures increases together with higher body weight reaching significant percentage in the breeding flock at final stages of the production cycle [25-30]. Experimental data from morphometric and mechanical studies on turkeys suffering from femoral fractures indicated an insufficient skeletal adaptation to heavy body weight as a causative factor responsible for locomotory function impairments [30]. Radiological analysis of tibiotarsal bone in six-week-old Pekin domestic duck has shown that lowered bone mass was associated with bone deformities and fractures [31,32]. Studies on broiler chickens from 15 different flocks revealed the presence of gross lesions in epiphysis and metaphysis of the proximal and distal femur. Moreover, bone fractures and separation of articular cartilage of the femoral bone head with progressive erosions of the subchondral bone were diagnosed confirming impaired bone formation and mineralization processes during skeletal development [33]. Decreased vBMD in proximal metaphysis between 2^nd^ and 4^th^ week, and in the diaphysis between 2^nd^ and 6^th^ week of posthatching development was observed in male Ross broiler chickens. These observations were associated with decreasing relative bone weight from 1.03% to 0.79% and tibia deformations and fractures occurrence [34]. Negative correlation between body weight and trabeculae number in tibiotarsal bone was found in growing domestic geese together with decreasing relative bone mass between 4^th^ and 16^th^ week of age. The observed negative changes of the trabecular structure combined with intensive body weight gain were suggested to be associated with bone deformities and locomotory impairments in those geese [35]. Femoral fracture incidence was also reported in heavy male turkey breeders at the age of 32–35 weeks [29]. Experimental studies on meat-type turkeys have shown that leg deformities are observed in birds characterized by decreased mechanical endurance and volumetric bone mineral density of tibia and femur [36]. In other studies on turkey model, it was shown that increased volumetric bone mineral density of femur and tibia contributes to higher values of maximum elastic strength and ultimate strength – the parameters representing mechanical endurance of bones [37].

Considering importance of intestinal bioavailability and absorption of phosphorus and its contribution to hydroxyapatite structure within bone tissue, the current study was performed to evaluate effects of administration with four different levels of *Escherichia coli* phytase on mineral density, morphology and mechanical endurance of femur in 16-week old turkeys. The results obtained in this study have shown that the most beneficial effects of phytase administration to growing turkeys was observed in group III receiving 125 FTU/kg of the diet. In this group, femur was characterized by increased geometric parameters such as cross-sectional area, second moment of inertia, mean relative wall thickness and cortical index when compared to the positive and negative control groups. The observed 5% insignificant increase of the volumetric bone mineral density in cortical bone of the midshaft in these birds was associated with significantly higher maximum elastic strength increased by 36% but cortical bone area decreased by 18.4% when compared to the positive control group. Similar effects of phytase administration on Cd, Wy and CBA of femur was observed in group IV that received 250 FTU/kg of the diet; however, all the differences of these parameters were significant in comparison to the positive control group and reached 9%, 44.6% and 21%, respectively. The effects of phytase administration in the dosage of 500 FTU/kg of the diet improved Cd, length and volume of femur with simultaneous reduction of CBA when compared to the positive control group. It is surprising that the analysis of femur obtained from the turkeys receiving the highest phytase dosage in this experiment has shown increased Ix value only in comparison to the positive control group, with concurrently lower CBA. In the negative control group, excluding significantly decreased value of CBA, the values of the analyzed parameters of femur were not significantly different from those obtained in the positive control group. Thus, simultaneous reduction of calcium and available phosphorus content in the turkey diet has not shown significant effects on morphological, densitometric and mechanical properties of femur. However, reduced dietary calcium and phosphorus content induced a significant decrease of final body weight of turkeys by 6.5%. It is worth to underline that both the initial and final body weights of turkeys administered with *Escherichia coli* phytase (Groups III – VI) were not significantly different from these parameters determined in the positive control group.

The results obtained from femur analyses show similarities with previous report describing effects of *Escherichia coli* phytase administration in the same experimental design on tibia properties [38]. In the previous report, ash content and specific gravity of whole tibia, as well as its maximum bone breaking force were not influenced by phytase administration at four different dosages. These observations correspond with the parameters analyzed for whole femur such as weight, length, total volume, MvBMD and Wf. However, contrary to the data from tibia obtained in the previous study, more detailed analyses of femur have shown positive effects of phytase administration in turkeys on geometric parameters and Cd assessed at the midshaft, and maximum elastic strength [38]. The results obtained in the current study are in accordance with previous experiments on male turkeys where *Escherichia coli* phytase administration at 250, 500, 750 and 1000 U/kg improved ash percentage and percentage of non-phytate phosphorus in middle toe and tibia in comparison to the control group. There were no statistically confirmed differentiated effects of the dosage applied on tibia and toe ash in these birds. However, *Escherichia coli* phytase was more effective when compared to two other commercially available phytases which were administered to turkeys [39]. The differences between the current and the previous study on turkeys results from its diverse phytase administration period and the applied dosages, as well as different age of birds and methodological approach. In the study by Applegate et al. (2003) the phytase administration was performed between 10^th^ and 21^st^ day of life when the experiment was terminated. Furthermore, neither morphometric and densitometric parameters nor mechanical endurance of the bones were analyzed in that experiment [39]. In studies on broiler chickens growing up to 49^th^ day of life, the inclusion of phytase at the dosage of 600 U/kg of diet has not induced significant effects on body, femur and tibia weights, as well as ash content in these bones. Moreover, densitometric analysis of whole body and tibia using DEXA method has not proven any effects of phytase administration on bone mineral density and bone mineral content [40]. The discrepancy of the results obtained in the experiment performed by Angel and colleagues (2006) and in the current study may be explained by different origin, treatment duration and dosage of the administered phytase, as well as age- and species-related differences. Among the mechanisms responsible for the observed beneficial effects of phytase administration on femur properties in this study, the improved health status of intestine and digestion processes, and better mineral absorption may be postulated. This hypothesis was confirmed in studies on turkeys in which phytase administration was associated with more efficient mineral, nitrogen and amino acid metabolism [5,41].

## Conclusion

In conclusion, the performed study has shown the most advantageous effects of *Escherichia coli* phytase administration on geometric, densitometric and mechanical properties of femur in turkeys receiving 125 and 250 FTU/kg of the diet. Phytase administration in all the experimental groups was associated with significantly decreased cortical bone area in the midshaft of femur. The reduced dietary calcium and phosphorus content in the diet of the negative control group of turkeys has significantly decreased the final body weight of turkeys by 6.5%. Phytase administration at the dosages of 500 and 1000 FTU/kg of the diet improved final body weight of turkeys in this study.

## Abbreviations

P: Phosphorus
Ca: Calcium
QCT: Quantitative computed tomography
vBMD: Volumetric bone mineral density
Td: Trabecular bone mineral density
Cd: Cortical bone mineral density
CBA: Cortical bone area
Bvol: Total bone volume
MvBMD: Mean volumetric bone mineral density
A: Cross-sectional area
Ix: Second moment of inertia
MRWT: Mean relative wall thickness
CI: Cortical index
Wy: Maximum elastic strength
Wf: Ultimate strength
ANOVA: One-way analysis of variance
DEXA: Dual-energy X-ray absorptiometry
